# Impact of national drug price negotiation policy on the accessibility and utilization of PCSK9 inhibitors in China: an interrupted time series analysis

**DOI:** 10.1186/s12939-024-02208-1

**Published:** 2024-06-05

**Authors:** Lingli Zhang, Xiaoye Wang, Hongting Yao, Baolong Ding, Xingyuan Gao, Xin Li

**Affiliations:** 1 School of International Pharmaceutical Business, China Pharmaceutical University, Nanjing, China; 2 School of Health Policy and Management, Nanjing Medical University, Nanjing, China; 3 School of Pharmacy, Nanjing Medical University, Nanjing, China; 4 Simcere Zaiming Pharmaceutical Co., Ltd., Nanjing, China; 5 Center for Global Health, School of Public Health, Nanjing Medical University, Nanjing, China

**Keywords:** Accessibility, Alirocumab, Evolocumab, National drug price negotiation policy, PCSK9 inhibitors, Utilization

## Abstract

**Background:**

PCSK9 inhibitors are a novel class of lipid-lowering drugs that have demonstrated favorable efficacy and safety. Evolocumab and alirocumab have been added to China’s National Reimbursement Drug List through the National Drug Price Negotiation (NDPN) policy. This study aims to evaluate the impact of the NDPN policy on the utilization and accessibility of these two PCSK9 inhibitors.

**Methods:**

The procurement data of evolocumab and alirocumab were collected from 1,519 hospitals between January 2021 and December 2022. We determined the monthly availability, utilization, cost per daily defined dose (DDDc), and affordability of the two medicines. Single-group interrupted time series (ITS) analysis was performed to assess the impact of the NDPN policy on each drug, and multiple-group ITS analysis was performed to compare the differences between them.

**Results:**

The NDPN policy led to a significant and sudden increase in the availability and utilization of PCSK9 inhibitors, along with a decrease in their DDDc. In the year following the policy implementation, there was an increase in the availability, utilization, and spending, and the DDDc remained stable. The affordability of PCSK9 inhibitors in China have been significantly improved, with a 92.97% reduction in out-of-pocket costs. The availability of both PCSK9 inhibitors was similar, and the DDDc of alirocumab was only $0.23 higher after the intervention. The market share of evolocumab consistently exceeded that of alirocumab. Regional disparities in utilization were observed, with higher utilization in the eastern region and a correlation with per capita disposable income.

**Conclusions:**

The NDPN policy has successfully improved the accessibility and utilization of PCSK9 inhibitors in China. However, regional disparities in utilization indicate the need for further interventions to ensure equitable medicine access.

**Supplementary Information:**

The online version contains supplementary material available at 10.1186/s12939-024-02208-1.

## Background

Cardiovascular diseases (CVDs) present a significant and growing challenge to global public health. The number of people with CVDs worldwide nearly doubled, rising from 271 million in 1990 to 523 million in 2019 [1]. In 2021, CVDs caused 20.5 million deaths, accounting for approximately one-third of all global mortality, a substantial increase from the 12.1 million CVDs-related deaths reported in 1990 [2]. In China, the prevalence of CVDs is rapidly increasing due to the aging population and changes in lifestyle. In 2020, there were approximately 330 million CVDs patients. CVDs are also the leading causes of death in China, accounting for 48.00% of deaths in rural regions and 45.86% in urban areas in 2020 [3].

Elevated blood lipid levels, particularly low-density lipoprotein cholesterol (LDL-C), are major risk factors for atherosclerotic CVDs, including myocardial infarction and stroke [4]. Proprotein convertase subtilisin/kexin type 9 (PCSK9) inhibitors, a new class of lipid-lowering drugs, have been proven to effectively reduce LDL-C levels, thereby significantly reducing the risk of CVDs [5]. PCSK9 inhibitors can provide an alternative treatment to statins for patients, particularly those who are statin intolerant or those who do not achieve their therapeutic goals with high-intensity statin therapy, such as patients with familial hypercholesterolemia. However, their high cost has historically hindered their widespread adoption, limiting access to this novel therapy for patients [6].

To reduce the prices of high-cost innovative drugs and enhance their accessibility, the Chinese government has implemented the National Drug Price Negotiation (NDPN) policy since 2017 [7]. The NDPN policy aims to utilize the collective bargaining power of the country to drive down the prices of high-priced drugs. According to the NDPN policy, price negotiation is a prerequisite for the inclusion of high-priced drugs in the National Reimbursement Drug List (NRDL). In 2021, when the NDPN policy was implemented, the Chinese government included all PCSK9 inhibitors available in China at that time, namely evolocumab and alirocumab, in the NRDL [8]. In contrast to many western countries where medicines are primarily dispensed in community pharmacies, in China, the majority of prescription drugs are dispensed in hospital pharmacies rather than community pharmacies. Regarding the PCSK9 inhibitors, approximately 85% of them are dispensed in hospital pharmacies.

Although the NDPN policy helps to reduce the prices of high-cost drugs and may theoretically promote their clinical use, the implementation of other health policies such as zero-markup on drugs [9] and global budget payment system [10] in the real world may hinder achieving this goal. Previous studies have demonstrated that the NDPN policy has enhanced the availability, utilization, and affordability of anticancer drugs, while also reducing their costs [11, 12]. However, its effects on PCSK9 inhibitors and other drugs remain unclear. Further research is needed to explore this impact and expand knowledge on the policy’s effects across different therapeutic areas. Moreover, as these two PCSK9 inhibitors share the same target and were added to the NRDL simultaneously, a comparison between the two drugs was feasible and of great interest.

Therefore, this study aims to evaluate the impact of the NDPN policy on the accessibility of PCSK9 inhibitors in China using interrupted time series (ITS) analysis. We will analyze the availability, utilization, cost, spengding, and affordability of evolocumab and alirocumab before and after the policy implementation. The findings will offer valuable insights for policymakers to optimize the NDPN policy and enhance the coverage and effectiveness of CVDs treatments.

## Methods

### Data collection

Continuous monthly procurement data was collected from hospitals using the Chinese Medicine Economic Information (CMEI) database. The database compiles procurement information from 1,519 hospitals nationwide, covering over 29.04% and 3.32% of tertiary and secondary hospitals in China, respectively. These hospitals are dispersed across 31 provincial administrative regions spanning the eastern, middle, and western regions of China. The collected data covered the timeframe from January 2021 to December 2022, during which the two PCSK9 inhibitors were included in the NRDL in January 2022. Specifically, data was gathered for a duration of 12 months both prior to and following the intervention.

The fundamental data on the two PCSK9 inhibitors was obtained from the National Healthcare Security Administration (NHSA) of China [13], and defined daily dose (DDD) were obtained from the World Health Organization (WHO) [14]. Additionally, information on the prevalence of dyslipidemia [15], population size, and per capita disposable income [16] in different regions was gathered. Cost data was reported in US dollars using the exchange rate of US$1 = 6.7328 Chinese yuan (CNY) [17].

### Outcome measures

Five main outcome measures were assessed: availability, utilization, cost, spending, and affordability. Methods for calculating these outcomes were developed based on the standard survey methodology developed by the World Health Organization and Health Action International (WHO/HAI) [18].

The availability of medicine was determined by dividing the number of hospitals that have purchased it by the total number of hospitals. Medicine utilization was measured using defined daily doses (DDDs) as recommended by the WHO [19]. DDDs were calculated by dividing the total volume procured by the defined daily dose (DDD), a metric that standardizes the dosage of a drug. The utilization in each region was also calculated. The cost of medicine was measured using cost per DDD (DDDc), which was calculated by dividing the procurement spending by DDDs.

The affordability of medicine was measured by the ratio of annual out-of-pocket (OOP) costs to the income that remains after meeting basic survival needs. If this ratio exceeded 40%, it was typically deemed a catastrophic health expenditure [20–22], indicating that the medicine was unaffordable and could potentially push the patient into poverty. Per capita disposable income was used to determine the income remaining after survival needs were met. The annual drug costs before and after the intervention were calculated by multiplying the average DDDc for the 12 months preceding and following the intervention by 365 days. The reimbursement rate for the two PCSK9 inhibitors included in the NRDL is 70%, with patients responsible for 30% of the OOP medicine cost. Patients are required to pay the total OOP cost for medicines not listed in the NRDL.

### Statistical analysis

We utilized ITS analysis to observe changes in the availability, utilization, DDDc, and spengding of two PCSK9 inhibitors. Initially, we conducted a single-group ITS to individually assess the impact of the NDPN policy on evolocumab and alirocumab, followed by a multiple-group ITS to compare the differences between the two.

The single-group ITS analysis was performed using the following regression model.

$$Yt=\beta0+\beta\mathit1Tt+\beta\mathit2Xt+\beta\mathit3XtTt+\varepsilon t$$

β0 is the intercept, β1 is the pre-intervention slope, β2 is the change in level that occurs immediately after the introduction of the intervention (relative to the counterfactual), β3 is the difference between the pre-intervention and post-intervention slopes.

The multiple-group ITS analysis was performed using the following regression model.

$$Yt=\beta\mathit0+\beta\mathit1Tt+\beta\mathit2Xt+\beta\mathit3XtTt+\beta\mathit4Z\;+\beta\mathit5ZTt+\beta\mathit6ZXt+\beta\mathit7ZXtTt+\varepsilon t$$

β0 to β3 represent the alirocumab group, similar to single-group ITS model; β4: difference in the intercept between evolocumab and alirocumab before intervention; β5: difference in the slope between evolocumab and alirocumab before intervention; β6: difference in level changes between evolocumab and alirocumab immediately after intervention start; β7: difference in slope between evolocumab and alirocumab post-intervention compared to pre-intervention [23].

In the two models above, Yt represents the aggregated outcome variable measured at time point t, Tt is the time since the start of the study, Xt is a dummy variable representing the intervention, and Z indicates the group (Z = 1 for the evolocumab and Z = 0 for the alirocumab). XtTt, ZTt, ZXt and ZXtTt are interaction terms.

Furthermore, the difference in post-intervention slopes between evolocumab and alirocumab was calculated using β5 + β7. We also calculated the difference in immediate post-intervention levels using the multi-group ITS models [24]. The coefficients were estimated using the Newey model with ordinary least squares (OLS) regression. Newey-West standard errors were produced to address autocorrelation and potential heteroskedasticity [25, 26].

One-way ANOVA was used to assess regional differences in medicine utilization. Pearson's correlation was used to examine the correlation between medicine utilization and the number of patients with dyslipidemia or per capita disposable income. Two-sided *p* < 0.05 was considered statistically significant. All data analyses were performed with Stata/MP V.16.0 software (StataCorp).

## Results

### Characteristics of medicines

Although both were globally marketed around the same time, evolocumab was introduced to the Chinese market over a year before alirocumab. In China, evolocumab has an additional approved indication compared to alirocumab. It can be used for homozygous familial hypercholesterolemia, in addition to reducing the risk of cardiovascular events and treating primary hypercholesterolemia and mixed dyslipidemia (Table 1).

**Table 1:** Characterization of two PCSK9 inhibitors

	Launch time in China	Global launch time	Marketing Authorization Holder	Indications approved in China	Defined daily dose
Evolocumab	2018.7	2015.7	Amgen	1. Reduction of the risk of cardiovascular events; 2. Primary hypercholesterolemia and mixed dyslipidemia; 3. Homozygous familial hypercholesterolemia	10 mg
Alirocumab	2019.12	2015.7	Sanofi-Aventis	1. Prevention of cardiovascular events; 2. Primary hypercholesterolemia and mixed dyslipidemia	5.4 mg

### Availability

**Fig. 1:**
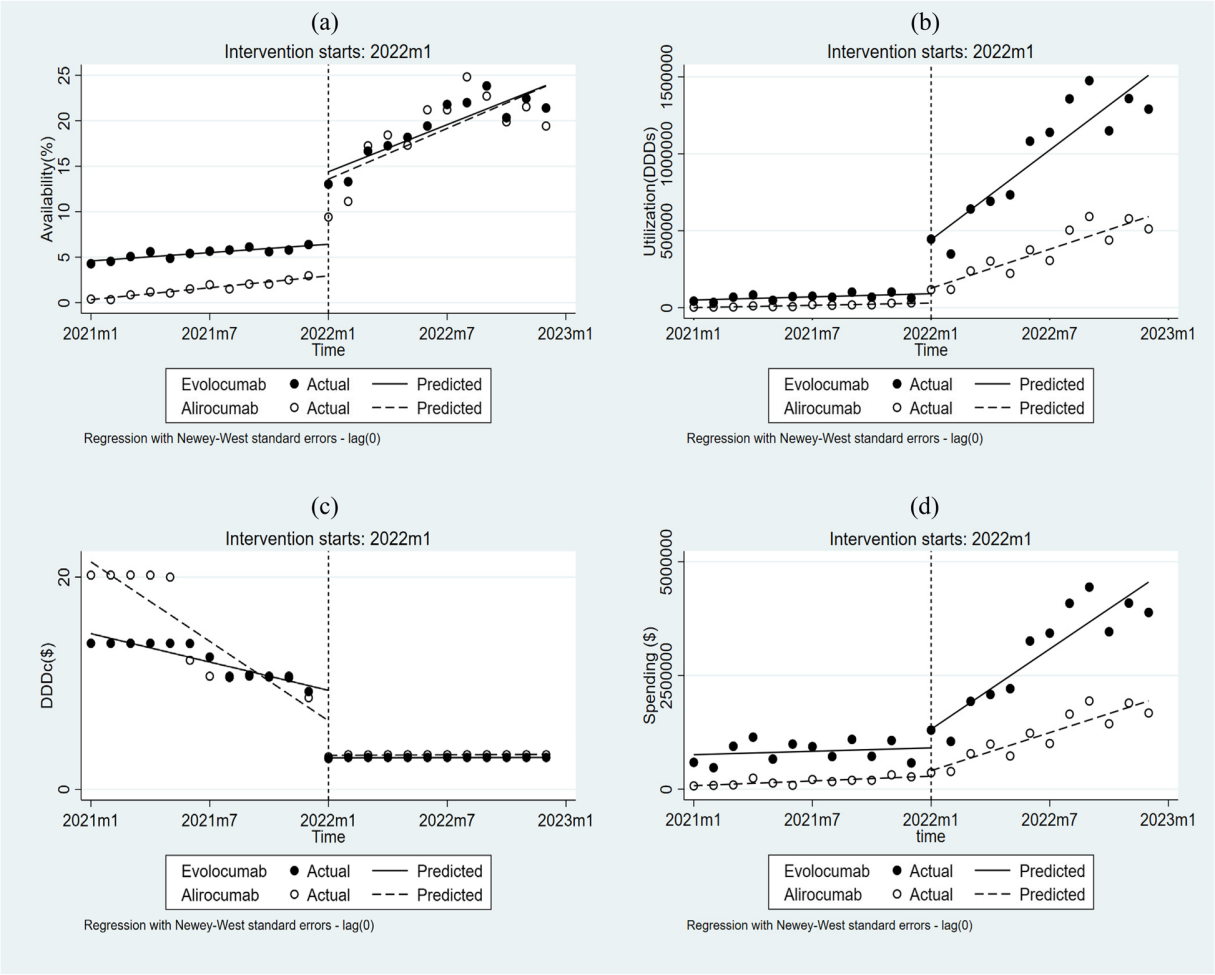
Observed and predicted availability (**a**), utilization (**b**), cost per daily defined dose (**c**) and spending (**d**) of two PCSK9 inhibitor

The availability of evolocumab increased by 7.97% (*p* < 0.001), and the availability of alirocumab increased by 10.63% (*p* < 0.001) immediately after the intervention (Table 2 and Fig. 1). The intervention also significantly increased the growth rate of availability of both evolocumab (β3 = 0.71, *p* < 0.001) and alirocumab (β3 = 0.71, *p* = 0.029).

**Table 2:** Changes in levels and trends of availability, utilization, cost, and spending

	Evolocumab			Alirocumab		
	Coefficient	*P* value	95%CI	Coefficient	*P* value	95%CI
**Availability (%)**						
Baseline line (β0)	4.58	< 0.001	4.22 to 4.95	0.33	0.002	0.14 to 0.53
Baseline trend (β1)	0.15	< 0.001	0.10 to 0.21	0.22	< 0.001	0.18 to 0.25
Level change immediately after intervention (β2)	7.97	< 0.001	6.20 to 9.73	10.63	< 0.001	6.65 to 14.61
Trend change after intervention (β3)	0.71	< 0.001	0.38 to 1.04	0.71	0.029	0.08 to 1.35
**Utilization (DDDs)**						
Baseline line (β0)	50,008.18	< 0.001	30,845.26 to 69,171.10	727.92	0.677	-2864.41 to 4320.25
Baseline trend (β1)	3411.30	0.049	10.26 to 6812.35	2449.93	< 0.001	1832.91 to 3066.96
Level change immediately after intervention (β2)	350,877.70	< 0.001	206,395.20 to 495,360.20	96,231.69	0.001	41,731.98 to 150,731.40
Trend change after intervention (β3)	93,745.91	< 0.001	65,071.36 to 122,420.50	39,885.30	< 0.001	28,842.23 to 50,928.36
**Cost per DDD (US$)**						
Baseline line (β0)	14.68	< 0.001	13.85 to 15.51	21.44	< 0.001	19.34 to 23.54
Baseline trend (β1)	-0.45	< 0.001	-0.55 to -0.34	-1.25	< 0.001	-1.49 to -1.00
Level change immediately after intervention (β2)	-6.36	< 0.001	-7.10 to -5.62	-3.26	0.002	-5.15 to -1.37
Trend change after intervention (β3)	0.45	< 0.001	0.34 to 0.56	1.25	< 0.001	1.01 to 1.50
**Spengding (US$)**						
Baseline line (β0)	757,715.30	< 0.001	466,437.50 to 1,048,993	75,675.14	0.008	21,816.53 to 129,533.70
Baseline trend (β1)	12,455.24	0.577	-33,363.38 to 58,273.86	17,426.96	< 0.001	10,296.34 to 24,557.59
Level change immediately after intervention (β2)	409,949.20	0.126	-125,454.80 to 945,353.20	121,596.60	0.187	-64,015.42 to 307,208.60
Trend change after intervention (β3)	281,775.20	< 0.001	184,529.30 to 379,021.10	122,021.10	< 0.001	85,054.90 to 158,987.40

*DDD* Defined daily dose

The initial availability of evolocumab was significantly higher than that of alirocumab (β4 = 4.25, *p* < 0.001, Table 3), and the growth rate of evolocumab availability was significantly lower than that of alirocumab before the intervention (β5 = -0.06, *p* = 0.037). After the intervention, the immediate availability of evolocumab was not significantly different from that of alirocumab (*p* = 0.697), and the upward trend between the two was not significantly different (*p* = 0.845).

**Table 3:** Differences in levels and trends between evolocumab and alirocumab

	Coefficient	*P* value	95%CI
**Availability (%)**			
Difference in baseline level (β4)	4.25	< 0.001	3.85 to 4.65
Difference in baseline trend (β5)	-0.06	0.037	-0.12 to 0.00
Difference in the immediate level change after intervention (β6)	-2.67	0.209	-6.88 to 1.55
Difference in trend change after intervention (β7)	0.00	0.994	-0.69 to 0.69
Difference in trend after intervention (β5 + β7)	-0.07	0.845	-0.76 to 0.62
Difference in the immediate level after intervention	0.81	0.697	-3.38 to 5.01
**Utilization (DDDs)**			
Difference in baseline level (β4)	49,280.26	< 0.001	30,390.01 to 68,170.5
Difference in baseline trend (β5)	961.37	0.565	-2387.67 to 4310.41
Difference in the immediate level change after intervention (β6)	254,646.00	0.001	105,030 to 404,262.1
Difference in trend change after intervention (β7)	53,860.61	0.001	24,088.96 to 83,632.26
Difference in trend after intervention (β5 + β7)	54,821.98	< 0.001	25,239.30 o 84,404.67
Difference in the immediate level after intervention	315,462.70	0.001	168,179.70 o 462,745.70
**Cost per DDD (US$)**			
Difference in baseline level (β4)	-6.76	< 0.001	-8.95 to -4.57
Difference in baseline trend (β5)	0.80	< 0.001	0.54 to 1.06
Difference in the immediate level change after intervention (β6)	-3.09	0.003	-5.06 to -1.13
Difference in trend change after intervention (β7)	-0.81	< 0.001	-1.07 to -0.54
Difference in trend after intervention (β5 + β7)	0.00	0.590	-0.02 to 0.01
Difference in the immediate level after intervention	-0.23	< 0.001	-0.34 to -0.13
**Spending (US$)**			
Difference in baseline level (β4)	682,040.10	< 0.001	395,039.20 to 969,041.00
Difference in baseline trend (β5)	-4971.73	0.824	-49,899.45 to 39,956.00
Difference in the immediate level change after intervention (β6)	288,352.60	0.295	-260,685.20 to 837,390.40
Difference in trend change after intervention (β7)	159,754.10	0.003	58,955.41 to 260,552.80
Difference in trend after intervention (β5 + β7)	154,782.40	0.001	64,550.02 to 245,014.80
Difference in the immediate level after intervention	910,732.00	< 0.001	465,793.10 to 1,355,671.00

*CI* Confidence interval, *DDD* Defined daily dose

### Utilization

As shown in Table 2 and Fig. 1, the intervention led to an immediate and significant increase in the utilization of both evolocumab (β2 = 350877.7, *p* < 0.001) and alirocumab (β2 = 96231.69, *p* < 0.001). The intervention also contributed to the increasing in the utilization of evolocumab (β3 = 93745.91, *p* < 0.001) and alirocumab (β3 = 39885.30, *p* < 0.001) in the following year.

Before the intervention, the utilization of evolocumab was significantly higher than that of alirocumab (β4 = 49280.26, *p* < 0.001), but the slope between the two was not significantly different (*p* = 0.565, Table 3). In the first month after the intervention, the utilization of evolocumab remained significantly higher than alirocumab at 315462.70 DDDs (*p* = 0.001), and the trend of evolocumab’s utilization was significantly higher than that of alirocumab (*p* < 0.001), with an average of 54821.98 DDDs higher per month post-intervention.

### Cost per DDD

The intervention resulted in an immediate decrease of $6.36 (*p* < 0.001) in the DDDc of evolocumab and $3.26 (*p* = 0.002) in the DDDc of alirocumab. The DDDc of both medicines remained stable thereafter (Table 2 and Fig. 1).

Before the intervention, the DDDc of alirocumab was significantly higher than that of evolocumab (β4 = -6.76, *p* < 0.001), and the decrease in DDDc of alirocumab exceeded that of evolocumab (β5 = 0.80, *p* < 0.001). In the first month after the intervention, the difference in DDDc between the two medicines narrowed, with alirocumab being only $0.23 higher than evolocumab (Table 3 and Fig. 1). The difference in the slope of DDDc after the intervention was not significant (*p* = 0.059).

### Spending

The spending on evolocumab (*p* = 0.126) and alirocumab (*p* = 0.187) did not change significantly at the first month of the policy implementation. However, spending on evolocumab and alirocumab increased significantly (*p* < 0.001) over time.

A comparison of the two drugs revealed that spending on evolocumab was significantly higher than that on alirocumab at the baseline (β4 = 682040.10, *p* < 0.001). Similarly, at the immediate intervention, spending on evolocumab was also significantly higher than that on alirocumab ($910732.00, *p* < 0.001). The spending on evolocumab exhibited a more pronounced increase than alirocumab following the intervention (*p* < 0.001).

### Affordability

The OOP cost for the two PCSK9 inhibitors decreased by 92.97% after the price reduction and reimbursement. Before the NDPN policy, the affordability ratio was 89.32%, which greatly exceeded catastrophic health expenditures. However, after the NDPN policy, the affordability ratio decreased to 6.25%, which was well below catastrophic health expenditures (Table 4).

**Table 4:** Affordability of PCSK9 inhibitor before and after NDPN

	Before NDPN		After NDPN		Reduction in out-of-pocket
	Annual out-of-pocket (US$)	Affordability ratio	Annual out-of-pocket (US$)	Affordability ratio	
Evolocumab	4464.25	81.49%	328.77	6.00%	92.64%
Alirocumab	5321.70	97.14%	356.49	6.51%	93.30%
Mean	4892.97	89.32%	342.63	6.25%	92.97%

*NDPN* National Drug Price Negotiation

### Market share

Evolocumab consistently held a higher market share than alirocumab throughout the observation period. Although the market share of alirocumab showed a slow increase, it only accounted for a maximum of 32.24% of spending and 33.44% of volume (DDDs) in the sample hospitals (Fig.S1 in Additional file 1).

### Disparities across regions

**Fig. 2:**
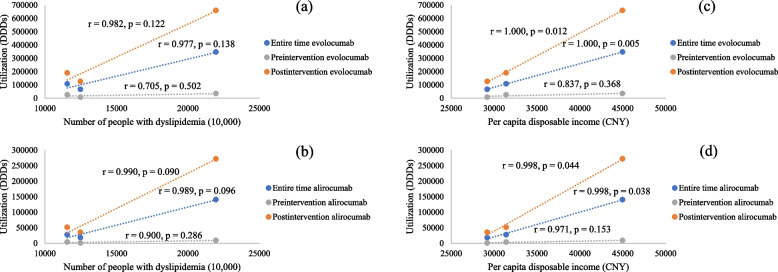
Correlation analysis of the utilization of evolocumab and alirocumab in various regions (**a**) between the number of patients and the utilization of evolocumab, (**b**) between the number of patients and the utilization of alirocumab; (**c**) between per capita disposable income and the utilization of evolocumab; (**d**) between per capita disposable income and the utilization of alirocumab

The utilization of evolocumab and alirocumab was significantly higher in the eastern region compared to the central and western regions (*p* < 0.001, Fig.S2 in Additional file 1). The utilization of both medicines was not correlated with the number of dyslipidemia cases in each region, either before or after the intervention. However, the average utilization of both medicines throughout the entire period or post-intervention was significantly correlated with per capita disposable income in each region (Fig. 2 and Table S1 in Additional file 1).

## Discussion

The NDPN policy led to a significant and sudden increase in the availability and utilization of PCSK9 inhibitors, while also decreasing their DDDc. In the year following the policy implementation, availability and utilization continued to increase, while DDDc remained stable. The gap in availability and DDDc between evolocumab and alirocumab, which existed prior to the intervention, was narrowed as a result of the policy. The availability of both PCSK9 inhibitors was similar, and the DDDc of alirocumab was only $0.23 higher after the intervention. The market share of evolocumab consistently exceeded that of alirocumab. The NDPN policy also substantially improved the affordability of PCSK9 inhibitors. Furthermore, we observed disparities in utilization across regions that were not related to prevalence but rather to per capita disposable income.

Controlling the cost and improving the accessibility of medicines is a topic of concern for policymakers. The Chinese government has attempted to address this issue through the NDPN policy. Previous studies have confirmed that the NDPN policy led to sharp increases in procured volumes and significant decreases in DDDc of anticancer medications [11, 12], which is consistent with our findings. However, previous studies have primarily focused on the impact of the NDPN policy on anticancer drugs. It is unclear whether this policy could have a similar effect on other drugs. Our study is the first to examine the impact of the NDPN policy on PCSK9 inhibitors.

PCSK9 inhibitors were more effective in reducing LDL-C levels and improving clinical outcomes than other lipid-lowering agents [27, 28]. However, in general, PCSK9 inhibitors were not cost-effective at their initial prices [29], which hindered their widespread adoption. We identified that the NDPN policy was highly effective in reducing costs and promoting utilization and availability. Both the immediate and long-term effects of the intervention were evident. Despite the reduction in price, our study demonstrated that spending on PCSK9 inhibitors increased one year after the policy was implemented, with a notable increase in utilization. Additionally, previous research has indicated that Chinese patients prioritize factors such as efficacy, safety, and mode of administration over OOP cost when selecting lipid-lowering drugs [30]. This change in priorities can be attributed to the NDPN policy, which has lowered the OOP cost of PCSK9 inhibitors.

This study also compared the availability, utilization, cost, and spending of the two drugs included in the NRDL at the same time. This comparison was not previously included in studies examining the impact of the NDPN policy. The availability and DDDc of two PCSK9 inhibitors were close after the intervention, but the utilization and spending of evolocumab consistently exceeded that of alirocumab before and after the intervention. This difference may be attributed to several factors. Firstly, evolocumab has exclusive approval for homozygous familial hypercholesterolemia, providing an additional indication compared to alirocumab. Secondly, as the first PCSK9 inhibitor approved in China, evolocumab has a first-mover advantage in the market. Thirdly, evolocumab has a cost advantage over alirocumab, with a DDDc $0.23 lower, which may result in higher utilization. Additionally, current evidence does not provide a clear advantage for either option. For instance, a meta-analysis of clinical trials indicated no significant differences in efficacy endpoints when excluding heterogeneity in the studied populations [31]. A meta-analysis demonstrated that evolocumab 140 mg biweekly was more effective than alirocumab 75 mg biweekly and 150 mg biweekly in reducing LDL-C [32]. Another meta-analysis suggested that alirocumab may provide optimal benefits for all-cause mortality with relatively fewer serious adverse events, while evolocumab may provide optimal benefits for myocardial infarction in patients with a high risk of cardiovascular events [33]. Therefore, further head-to-head trials with long-term follow-up and high methodological quality are necessary to compare the two PCSK9 inhibitors.

Our study observed regional disparities in the utilization of PCSK9 inhibitors that were not correlated with the prevalence of dyslipidemia but were associated with per capita disposable income. When disparities in drug utilization were identified across regions, it was anticipated that PCSK9 inhibitors would be utilized more frequently in regions with a higher number of dyslipidemia patients. However, this was not observed. For instance, the western region had a greater number of patients with dyslipidemia than the central region, yet exhibited lower utilization than the central region. Instead, the study revealed a correlation between the utilization of these drugs and disposable income per capita. This finding suggests that socioeconomic factors play a critical role in medicine utilization and highlights the persistent challenge of health inequities. Therefore, targeted interventions are necessary to address these disparities and ensure equitable distribution of benefits from the NDPN policy across different regions.

This research has several limitations. Firstly, the annual costs were calculated without considering patient discontinuation, potentially leading to an overestimation of annual costs. Secondly, due to the absence of procurement data from community pharmacies, this study only analyzed data from hospitals. However, PCSK9 inhibitors are prescription drugs, and in China’s health system, hospitals are the primary source for obtaining them. Thirdly, we only examined the correlation between prevalence and per capita disposable income with PCSK9 inhibitor utilization. Future research should be conducted to understand the underlying factors contributing to these regional disparities.

## Conclusions

The NDPN policy has increased the availability and utilization of PCSK9 inhibitors while reducing costs and improving affordability. The utilization of evolocumab was consistently higher than that of alirocumab. After the intervention, the DDDc of evolocumab was slightly lower than that of alirocumab, and there was no significant difference in the availability of the two. However, there were disparities in utilization across regions, indicating the need for additional strategies to address inequality.

### Supplementary Information

Additional file 1.

## Data Availability

No datasets were generated or analysed during the current study.
