# Supplementary material for: Impact of national drug price negotiation policy on the accessibility and utilization of PCSK9 inhibitors in China: an interrupted time series analysis
Source: Int J Equity Health. 2024 Jun 5;23:116. doi: 10.1186/s12939-024-02208-1 (PMC11154977; doi:10.1186/s12939-024-02208-1)
Supplement: Supplementary file 1 — Additional file 1. [file 12939_2024_2208_MOESM1_ESM.docx]

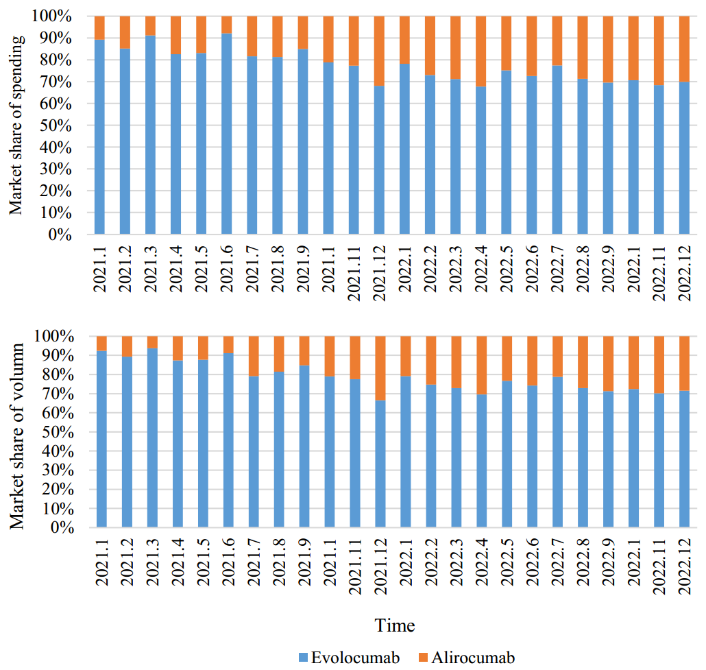


**Fig**.S1 Market share of evolocumab and alirocumab


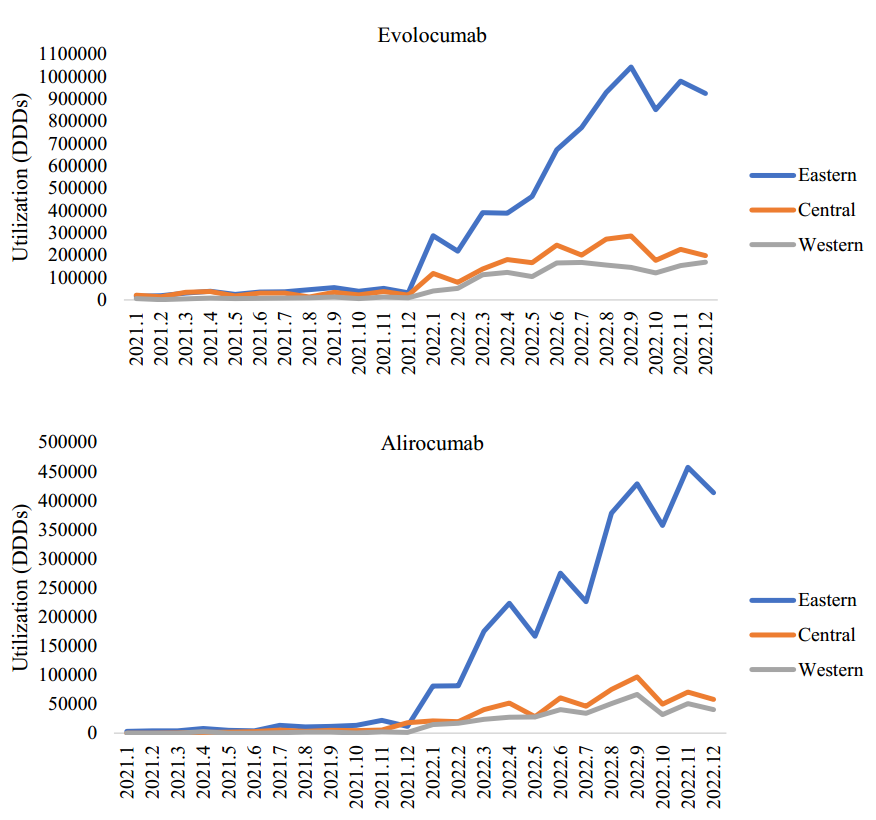


**Fig.S2** Utilization of evolocumab and alirocumab in different regions

**Table S1** Pevalence, per capita disposable income, and utilization in different regions

|  | **Western** | **Central** | **Eastern** |
| --- | --- | --- | --- |
| Prevalence of dyslipidemia (%)[1] | 32.58 | 31.70 | 33.16 |
| Total population (10 000) [2] | 38306.00 | 36456.00 | 66213.00 |
| Number of patients (10 000) | 12480.09 | 11556.55 | 21956.23 |
| Per capita disposable income (CNY) [2] | 29191.48 | 31400.14 | 44973.91 |
| **Utilization (DDDs)** |  |  |  |
| Entire time evolocumab | 66843.00 | 108182.08 | 347453.17 |
| Entire time alirocumab | 18276.00 | 27783.60 | 140643.00 |
| Preintervention evolocumab | 7652.17 | 25894.17 | 35224.00 |
| Postintervention evolocumab | 126033.83 | 190470.00 | 659682.33 |
| Preintervention alirocumab | 961.81 | 3991.90 | 9248.84 |
| Postintervention alirocumab | 35590.28 | 51575.23 | 272037.00 |

CNY, Chinese yuan; DDDs, defined daily doses.

**Reference**

1. Lu Y, Zhang H, Lu J, Ding Q, Li X, Wang X, et al. Prevalence of dyslipidemia and availability of lipid-lowering medications among primary health care settings in China. JAMA Netw Open. 2021;4:e2127573.

2. National Bureau of Statistics. China Statistical Yearbook-2023. https://www.stats.gov.cn/sj/ndsj/2023/indexch.htm.
